# The Burden of COVID-19 in the Immunocompromised Patient: Implications for Vaccination and Needs for the Future

**DOI:** 10.1093/infdis/jiad181

**Published:** 2023-08-04

**Authors:** Andrea Antinori, Mary Bausch-Jurken

**Affiliations:** Clinical and Research Infectious Diseases Department, National Institute for Infectious Diseases Lazzaro Spallanzani, IRCCS, Rome, Italy; Moderna, Inc., Cambridge, Massachusetts, USA

**Keywords:** COVID-19, immunocompromise, mRNA vaccine, vaccination, SARS-CoV-2

## Abstract

Approximately 3% of US adults are immunocompromised and less capable of fighting infections such as SARS-CoV-2 (the causative agent of COVID-19). Individuals may be immunocompromised for reasons related to an underlying medical condition or to immunomodulatory therapies that alter the immune response. In general, vaccination with mRNA–based vaccines is effective at reducing COVID-19–associated hospitalization and death among immunocompromised populations, particularly after 3 or more doses. However, the immunocompromised population is heterogeneous, with COVID-19 vaccine-elicited immune responses and risk for severe COVID-19 existing on a continuum. Therefore, understanding the impact of vaccination and the complexity of immune responses across heterogeneous immunocompromised individuals is essential for guiding effective vaccination regimens including additional (booster) doses. In this article, we provide an overview of the immunocompromised population and the burden of disease attributable to COVID-19, while discussing key opportunities and challenges of vaccinating immunocompromised individuals.

## THE IMMUNOCOMPROMISED POPULATION: A HETEROGENEOUS POPULATION AT RISK FOR POOR OUTCOMES WITH COVID-19

Approximately 3% of the adult population in the United States is immunocompromised [1] and less capable of fighting infections due to alterations in the immune system [2, 3]. Immunocompromising conditions can arise through a multitude of causes, and the underlying reason for immunosuppression can result in drastically different responses to infection and vaccination. Specifically, individuals can be considered immunocompromised for reasons related directly to an underlying medical condition or to immunomodulatory therapies that alter the immune system [4]. Medical conditions include primary and secondary immunodeficiencies. Primary immunodeficiencies are caused by genetic abnormalities affecting immune system cells such as common variable immunodeficiency disease, severe combined immunodeficiency, DiGeorge syndrome, and Wiskott-Aldrich syndrome [4–7]. Secondary immunodeficiencies develop from conditions that are extrinsic to the immune system but subsequently cause an impaired immune response. For example, AIDS is a secondary immunodeficiency caused by HIV infection, occurring in individuals living with advanced or untreated HIV infection or those who do not adequately respond to antiretroviral therapy [8]. Metabolic diseases such as diabetes and chronic kidney disease can also lead to secondary immunodeficiencies [8, 9]. In addition to these medical conditions, moderate to severe immunocompromise can result from immunosuppressive or immunomodulatory medications given to recipients of organ transplants, individuals with cancer, or those with autoimmune conditions [4].

Due to weakened immune responses, immunocompromised individuals are more susceptible to severe outcomes and complications of infectious disease [10–12]. Accordingly, preexposure vaccination remains critical to protect this population but is also challenging in regard to both safety and efficacy [2]. Live vaccines are typically not recommended for immunocompromised individuals due to potential safety risks, while immune responses elicited by recommended vaccines can be insufficient due to the impaired immune systems [10, 11, 13]. Vaccine efficacy can also vary based on intrinsic patient characteristics, such as the exact underlying disease and the type of immunosuppressive therapy, while also differing based on the dosing regimen and vaccine type [2].

**Figure 1.**
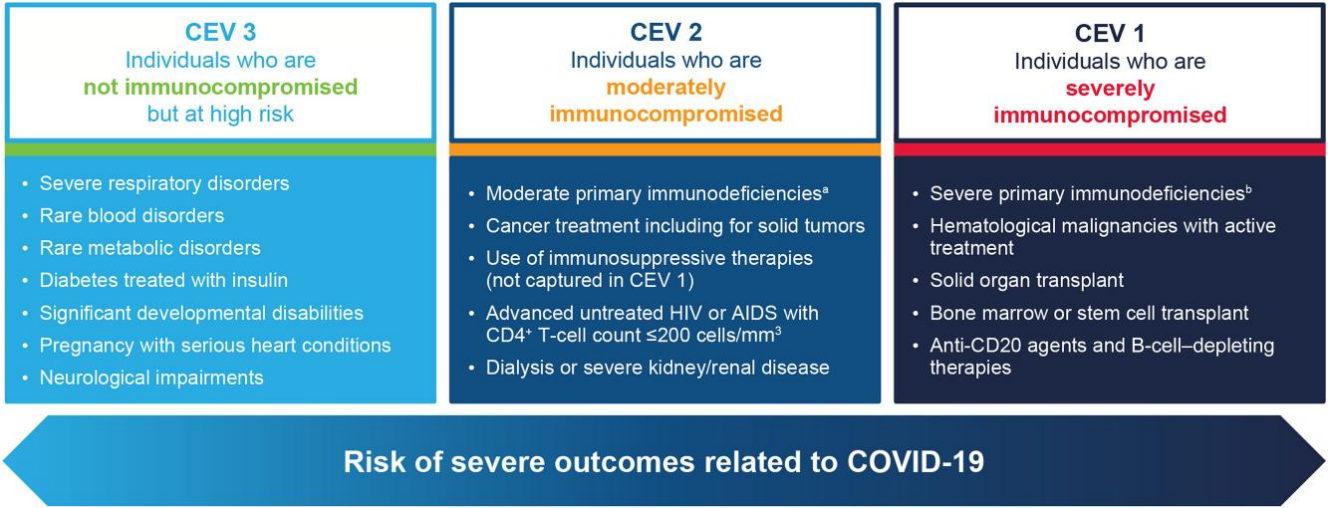
Spectrum of COVID-19 risk continuum for the clinically extremely vulnerable immunocompromised population. ^a^Moderate primary immunodeficiencies include conditions that require ongoing immunoglobulin replacement therapy or primary immunodeficiencies that have confirmed genetic causes (eg, DiGeorge syndrome and Wiskott-Aldrich syndrome). ^b^Severe primary immunodeficiencies include combined immunodeficiencies affecting T cells, immune dysregulation (particularly familial hemophagocytic lymphohistiocytosis), and type I interferon defects. Abbreviations: CEV, clinically extremely vulnerable.

The emergence of the SARS-CoV-2 that caused the global COVID-19 pandemic further underscored the vulnerability to infection and the complexity of vaccination in this population. Early in the pandemic, some countries developed criteria to assist with identifying those individuals at high risk of poor outcomes from COVID-19 who should be appropriately shielded from exposure (clinically extremely vulnerable [CEV]; Figure 1) [14, 15]. As COVID-19 vaccines became available, the CEV criteria were further used to prioritize individuals for initial vaccination, with these criteria now being used to prioritize individuals who may benefit from additional doses of COVID-19 vaccines as well as early therapies for COVID-19 once they become available [14]. Notably, COVID-19 vaccines offer protective immunity by helping reduce the symptoms of COVID-19 and protecting against severe outcomes of disease; they do not prevent infection.

An immune correlate of protection (CoP) is a marker that can be used to reliably predict the efficacy of a vaccine [16]. So far, no universal CoP has been defined for COVID-19 vaccines, but a growing body of evidence suggests that both anti–SARS-CoV-2 binding and neutralizing antibody titers correlate with vaccine efficacy [16–18]. However, cellular immune responses are also integral to immune memory, playing an important role in protection from severe disease, and is of interest among immunocompromised individuals who may not mount sufficient antibody responses following primary series vaccination [19–21]. As such, immunogenicity measurements are important indicators of how well a vaccine is likely to perform and can function as estimators of efficacy in the absence of a CoP.

Defining the clinical vulnerability for COVID-19 across the complex spectrum of immunocompromised individuals is challenging, and there remains detailed nuance to the burden of disease and response to vaccination among this population. As the COVID-19 pandemic continues and eventually moves into a more endemic state, there is an urgent need to further estimate the risks associated with COVID-19 disease and the complexities of vaccination in each disparate group of immunocompromised individuals. As such, this review aims to provide a high-level overview of the immunocompromised population and the burden of disease attributable to COVID-19, while discussing key opportunities and challenges of vaccinating immunocompromised individuals; additional articles within this supplement will go into more detail on specific immunocompromised populations.

## BURDEN OF COVID-19 IN IMMUNOCOMPROMISED POPULATIONS AND THE IMPACT OF VACCINATION

In contrast to immunocompetent individuals, who can generally mount a sufficient immune response against SARS-CoV-2, those with immune deficiencies may not and, thus, have an increased likelihood of COVID-19–associated morbidity and mortality [4, 22, 23]. At the population level, all individuals who are immunocompromised have a higher risk of hospitalization and poor outcomes related to COVID-19 [23, 24]. In a case-control study among adults, individuals broadly classed as immunocompromised had a 2.68-fold greater adjusted odds of being hospitalized with COVID-19 than immunocompetent individuals [25]. However, the burden of COVID-19 also varies substantially within the immunocompromised population. The exact mechanisms underlying susceptibility to severe COVID-19 are complex and vary based on the type of immunocompromising condition, the use of different immunosuppressive therapies, and the presence of other medical comorbidities, among other factors [26]. In a recent retrospective cohort study, severity outcomes from COVID-19 in individuals with and without preexisting autoimmune disease (AID) and/or exposure to immunosuppressants were evaluated. Individuals with preexisting AID only had a 1.13-fold (95% confidence interval [CI], 1.09- to 1.17-fold) increase in a life-threatening condition compared with those without a preexisting AID only; a 1.35-fold (95% CI, 1.29- to 1.40-fold) increase in a life-threatening condition was observed among those with preexisting AID taking immunosuppressants compared with those without a preexisting AID taking immunosuppressants [27]. Although there is strong evidence that individuals with particular immunocompromising conditions are at higher risk of severe COVID-19 outcomes [28], it is important to recognize that the risk of severe COVID-19 exists on a continuum for immunocompromised individuals (Figure 1). Although criteria such as CEV can offer broad guidance regarding clinical vulnerability, it is important to emphasize the heterogeneity that exists even among the same immunocompromising conditions. For example, consensus on risk is still lacking for certain groups, such as individuals living with HIV. Notably, despite some early pandemic studies showing no significant association between HIV and severe COVID-19—particularly among those achieving viral suppression through antiretroviral therapy [29, 30]—recent evidence has demonstrated a strong association between individuals with advanced untreated HIV and severe COVID-19 hospitalization and death [31–34].

In general, vaccination with mRNA–based vaccines (eg, mRNA-1273 [SPIKEVAX; Moderna, Inc., Cambridge, MA, USA] and BNT162b2 [COMIRNATY; Pfizer Inc., New York, NY, USA; BioNTech Manufacturing GmbH, Mainz, Germany]) is effective at reducing COVID-19–associated hospitalization among immunocompromised populations, particularly after 3 or more doses [35, 36]. However, once hospitalized with laboratory-confirmed COVID-19, even vaccinated immunocompromised individuals have higher odds of intensive care unit admission and in-hospital death than immunocompetent individuals [23]. Despite this, real-world evidence indicates that vaccinated immunocompromised individuals do have improved outcomes following breakthrough infection compared with unvaccinated immunocompromised individuals [37–39].

Not only does COVID-19 severity vary by immunocompromising condition, but the effectiveness of COVID-19 vaccination can also vary based on the exact immunocompromised population under investigation. In a multicenter, observational study in the United States, 2 doses of an mRNA-based vaccine had 29% effectiveness against COVID-19 hospitalization among solid organ transplant recipients (SOTRs), a severely immunosuppressed population, while effectiveness was 72% for patients with other immunocompromising conditions [36]. However, 3 doses substantially increased effectiveness to 77% for SOTRs and 92% for patients with other immunocompromising conditions [36]. Therefore, understanding the impact of vaccination and the overall complexity of immune responses across heterogeneous immunocompromised individuals is essential for guiding effective vaccination regimens including additional (booster) doses and other preventive strategies for this vulnerable population.

## FACTORS AFFECTING IMMUNE RESPONSE TO SARS-COV-2 VACCINATION IN THE IMMUNOCOMPROMISED POPULATION

Immune responses against SARS-CoV-2 involve complex synergy between both humoral- and cellular-mediated immunity [22, 40]. As such, immune responses to COVID-19 vaccination can vary widely among different immunocompromising conditions due to different alterations in the immune system. Although COVID-19 vaccine trials have largely excluded immunocompromised groups, observational studies have begun to shed light on the factors underlying these disparate immune responses as well as key challenges for vaccination in this population.

### The Level of Immunosuppression Affects Responses to Vaccination

A growing body of evidence has highlighted that the level of immunosuppression of an immunocompromised individual can play a role in vaccine-elicited immune responses. For example, a recent systematic review evaluated response to COVID-19 vaccination among individuals with immune-mediated inflammatory diseases (IMIDs); significantly lower seroconversion rates after a 2-dose regimen of an mRNA vaccine were observed in patients with IMIDs compared with healthy controls (odds ratio, 0.05) [41]. Disease-specific seroconversion rates were 79.5% among individuals with rheumatoid arthritis, 90.7% among those with systemic lupus erythematosus, 70.5% among those with vasculitis, 95.2% among those with inflammatory bowel disease, and 95.6% among those with spondyloarthropathy [41]. These findings highlight that the disease state among comparable populations can result in varied humoral responses to COVID-19 vaccination.

Variation in immune response following COVID-19 mRNA vaccination has also been demonstrated in individuals living with HIV. Although the impact of coinfection with HIV plus SARS-CoV-2 on clinical outcomes has yet to be fully elucidated, a recent study demonstrated that immune responses to COVID-19 vaccines may be related to the CD4^+^ T-cell count [42, 43]. In individuals living with HIV with CD4^+^ T-cell counts >500 cells/mm^3^, the humoral and cell-mediated immune responses following 2 doses of BNT162b2 or mRNA-1273 were comparable with HIV-negative control individuals who received 2 doses of BNT162b2. However, for those with CD4^+^ T-cell counts <200 cells/mm^3^, SARS-CoV-2 neutralizing activity was detected in approximately 70% of individuals, substantially lower than what was observed in individuals with high CD4^+^ T-cell counts (93.1%) and HIV-negative control individuals (98.6%) [43]. Interestingly, this same study highlighted that neutralizing antibody response to SARS-CoV-2 after vaccination was greater in individuals living with HIV than observed in a study in SOTRs, where only approximately 35% were estimated to develop neutralizing antibodies against SARS-CoV-2 [43, 44].

These findings emphasize that the immunocompromised population is heterogeneous and that COVID-19 vaccine–elicited immune responses as well as severe outcomes and complications of disease exist on a continuum. Additional articles in this supplement will go into greater depth on both immune responses and outcomes for specific immunocompromised populations.

### Immunosuppressive Therapy Affects Responses to Vaccination, Regardless of the Underlying Diagnosis

Evidence has also shown that the immunosuppressive therapies these individuals may be receiving are important contributors to the variable immune responses to COVID-19 vaccination. For example, regardless of underlying diagnosis, the risk for infection and hospitalization with COVID-19 in vaccinated individuals was increased when taking immunosuppressants [45, 46]. Moreover, individuals receiving immunosuppressive therapies had an increased risk of SARS-CoV-2 infection compared with immunocompetent individuals irrespective of vaccination status (fully vaccinated, adjusted hazard ratio [aHR], 2.17 [95% CI, 1.69–2.79]; unvaccinated, aHR, 1.40 [95% CI, 1.07–1.83]). Among fully vaccinated groups, the risk of hospitalization with COVID-19 was higher for those receiving immunosuppressants than those who did not (aHR, 4.86 [95% CI, 2.24–10.56]) [45].

The type of immunosuppressive therapy received can also greatly affect immune responses to COVID-19 vaccines. Known predictors of lower responses include the use of B-cell–depleting therapies; treatment with antimetabolites, abatacept, or Janus kinase inhibitors; and the long-term use of glucocorticoids at high doses [41, 47, 48]. The impact of other therapies is less clear, but anti–tumor necrosis factor therapies, mycophenolate mofetil, and methotrexate can also lead to lower seroconversion rates and lower SARS-CoV-2 anti–receptor-binding domain (RBD) immunoglobulin G (IgG) titers [41, 48]. B-cell–depleting therapies, in particular, can pose a major challenge for COVID-19 vaccination, as a reduction in peripheral B lymphocytes is associated with failure to seroconvert [49, 50]. Rituximab, an anti-CD20 B-cell–depleting therapy, has a known history of impairing immunogenicity of vaccines [51, 52] and has also been shown to diminish immune responses to COVID-19 vaccines. In a prospective cohort study, only 19 of 87 individuals (21.8%) with rheumatoid arthritis receiving rituximab compared with 1096 of 1114 immunocompetent individuals (98.4%) had a serological response to SARS-CoV-2 RBD following 2 doses of a COVID-19 mRNA vaccine (*P* < .0001) [51]. Importantly, although B-cell–depleting therapies can be problematic for vaccination, additional doses of COVID-19 vaccines can increase both humoral and cellular responses, as described in the next section [49].

### Additional Doses of COVID-19 Vaccines Can Improve Immune Responses in Immunocompromised Individuals

When mRNA-based COVID-19 vaccines first became available in the United States, it was recommended that immunocompromised individuals receive a 2-dose primary vaccination schedule. However, it has become clear that this 2-dose schedule may not adequately protect moderately or severely immunocompromised individuals. A 3-dose primary vaccination regimen of mRNA-based vaccines is now recommended for this population in both the United States and the European Union, with several European countries now recommending 2 additional doses to supplement the 3-dose primary vaccination of immunocompromised individuals [53, 54]. Among US adults, vaccine effectiveness against COVID-19–associated hospitalization has been shown to be 88% (95% CI, 81%–93%) in immunocompromised individuals who received 3 doses of mRNA-based vaccines compared with 69% (95% CI, 57%–78%) in those who received 2 doses (*P* < .001) [35].

A 3-dose regimen of mRNA-based COVID-19 vaccines has been shown to seroconvert individuals with immune-mediated inflammatory disorders in receipt of immunosuppressants [48]. In a cohort study, a greater percentage of individuals seroconverted after receiving a third dose compared with a second dose (anti-CD20 group, 45.6% vs 19.1%; mycophenolate mofetil group, 89.5% vs 37.8%; sphingosine 1-phosphate group, 48.4% vs 25.8%) [48]. As stated earlier, B-cell–depleting therapies such as rituximab are known to impair immune responses to vaccination among immunocompromised individuals. In individuals without prior exposure to SARS-CoV-2 with a treatment history of anti-CD20 drugs (rituximab or ocrelizumab), those who were humoral nonresponders after 2 vaccine doses (mRNA-1273, 8 of 32; BNT162b2, 23 of 32) were given a third dose, of which 6 of 32 seroconverted [55]. Following an additional dose (third dose of mRNA-1273 or BNT162b2; or second dose of Ad26.COV2.S) in individuals on anti-CD20 drugs, 50.0% (4 of 8) had an anamnestic (ie, enhanced) response, demonstrating that immunosuppressive effects can be partially overcome by administration of additional doses in some individuals [56]. As detectable B cells contribute to seroconversion rates in rituximab-treated individuals, current evidence supports delaying the third dose until B cells have repopulated or postponing rituximab treatment until after vaccination [49, 57].

Thus far, evidence also indicates that B-cell–depleting therapies do not affect the T-cell response, which may also be further enhanced by additional doses of COVID-19 vaccines and supports additional doses being prioritized in individuals in receipt of these therapies [49, 51]. In a longitudinal, prospective cohort study, all 12 rituximab-treated individuals showed robust T-cell responses following a third dose of a COVID-19 vaccine despite low serological response (∼20%) [51]. Several studies have further demonstrated that most nonresponders on rituximab will remain seronegative even after a third dose of mRNA-1273 or BNT162b2; some do respond with evidence of an improved cellular response [48, 51, 55, 56].

It has also been suggested that a third dose can increase spike-reactive B cells and CD4^+^ T cells in SOTRs [58, 59]. In a double-blind, randomized controlled trial, 55% of SOTRs given a third dose of mRNA-1273 had anti-RBD antibodies (≥100 U/mL) compared with 18% of SOTRs receiving placebo. Median T-cell counts specific to SARS-CoV-2 were also greater following a third dose of mRNA-1273 compared with placebo (432 vs 67 cells per 10^6^ CD4^+^ T cells, respectively) [58]. Furthermore, individuals with HIV who were in receipt of antiretroviral therapy at the time of the third dose showed strong increases in humoral and cellular response following a third COVID-19 mRNA vaccine dose with anti-RBD IgG neutralizing antibody titers greater than those observed 1 month after the 2-dose vaccination [60]. Of participants living with HIV with severe immunodeficiency, 95.5% showed robust anti-RBD IgG response, 86.3% had neutralizing antibody responses, and 70% had T-cell immunity following the third dose, without a significant association with the current level of CD4 count. Characteristically, the humoral response following the third dose was higher than achieved with the second dose, whereas cell-mediated immunity remained more stable after dose 2 compared with values achieved after dose 3 [60].

After the 3-dose primary series, an additional fourth dose administered at least 2 months after dose 3 is also recommended for immunocompromised populations. Currently, variant-updated booster vaccines are recommended as a fourth dose to potentially expand protection against emergent SARS-CoV-2 variants of concern. These bivalent vaccines are composed of both ancestral and an omicron variant strain, as studies have shown that the prototype mRNA-based vaccines containing only the ancestral SARS-CoV-2 strain (ie, mRNA-1273 and BNT162b2) elicit lower immune responses to the heavily mutated omicron strain in both immunocompetent and immunocompromised populations. For example, a recent observational cohort study in individuals with autoimmune rheumatic diseases (ARDs) demonstrated that antibody titers against the omicron (B.1.1.529) variant remained low despite a third dose; mean cross-neutralizing responses against omicron variant were lower in immunocompromised individuals with ARDs compared with healthcare workers (26.8% vs 50.3%, *P* < .0001) [61]. Although evaluations on the immune responses to bivalent omicron-containing vaccines among the immunocompromised population are still relatively limited, a recent study among individuals living with hematologic malignancies demonstrated that recipients of omicron-containing bivalent mRNA vaccines had an 89% relative risk reduction in COVID-19–related hospitalization and reduction in median duration of hospitalization compared with their nonvaccinated counterparts [62].

### Differential Immune Responses to Heterologous COVID-19 Vaccination Regimens

Heterologous or “mix-and-match” vaccination regimens have been implemented in certain countries to allow for different types of COVID-19 vaccines to be administered for the primary regimen and/or booster doses. These strategies have allowed for varied vaccine combinations and schedules in response to the rapidly emerging evidence on vaccine effectiveness and the availability of vaccine(s) for booster administration. Among immunocompromised individuals, the immune response to heterologous vaccine doses is dependent upon the initially administered vaccine [63–65]. Heterologous vaccination with additional doses of mRNA-based vaccines after completion of the primary regimen has been shown to increase the humoral response to other vaccine types in low responders to previous vaccination [63–67]. A third dose of mRNA-based vaccine (BNT162b2 or mRNA-1273) following a heterologous CoronaVac/ChAdOx1 2-dose schedule enhanced serological response in immunocompromised individuals with solid tumors (2330 BAU/mL [95% CI, 1487–3649]) to levels comparable with immunocompetent individuals (3823 BAU/mL [95% CI, 3446–4241]) despite low pre–third dose anti-RBD IgG titers [63]. Furthermore, in ChAdOx1- or BNT162b2-primed immunocompromised individuals on long-term dialysis, an additional dose of mRNA-1273 elicited significantly increased SARS-CoV-2–specific antibodies, with strong humoral responses (>500 BAU/mL) in 78.4% of individuals [67].

In a single-center, randomized, blinded study, immunocompromised individuals who did not seroconvert following 2 doses of an mRNA vaccine had increased seroresponse following a third homologous mRNA-based vaccine dose (15 of 24 [63%]) as opposed to a heterologous vector-based vaccine (4 of 22 [18%]); *P* = .006) [68]. However, the addition of a third dose of a viral vector-based vaccine dose following 2 doses of mRNA-based vaccine increased antibody titers in kidney transplant recipients from month 1 to month 3, compared with a third dose of homologous mRNA-based vaccine where titers remained unchanged; this resulted in lower antibody levels in the homologous vaccination group (mRNA, 25.8 U/mL; viral vector, 77.7 U/mL; *P* = .038) [69].

## CONCLUSIONS

Although individuals with weakened immune systems can be broadly classified as immunocompromised, the disparate immune responses to COVID-19 vaccination and outcomes among this population highlight the overall lack of homogeneity among this group. The underlying medical condition and the use of certain immunosuppressants can substantially affect immune responses to vaccination among immunocompromised individuals. As such, regulatory agencies have recommended extending the primary vaccination series to include 3 doses as well as the administration of additional (booster) doses of COVID-19 vaccines, which have been shown to increase humoral and/or cellular response in certain immunocompromised individuals with initially poor immune responses [49]. Although healthcare professionals and policy-makers have prioritized COVID-19 vaccination with a 3-dose regimen among the immunocompromised population, there is still a need to ensure that these patients remain up-to-date with the recommendations for additional doses, including bivalent vaccines. Moving forward, considerations for the subgroup of immunocompromised populations must remain a priority to protect this group from severe disease.

Knowledge of COVID-19 vaccine–elicited immunogenicity among this population is critical for guiding effective healthcare for these patients. Accordingly, understanding the current challenges and potential limitations of COVID-19 vaccination in this group is essential and can inform not only decisions on additional vaccine doses, but also recommendations for nonpharmaceutical preventive strategies as well as prophylaxis approaches. This also becomes particularly important as variants evolve, making monoclonals and prophylaxis minimally effective. Investing in ways to optimize protection for these individuals can significantly impact the course of this pandemic as prolonged infection can give rise to SARS-CoV-2 variants of concern with increased potential to escape vaccine- and natural immunity–mediated protection. Furthermore, as COVID-19 potentially transitions from a pandemic to an endemic disease, the establishment of effective healthcare measures remains a public health priority to protect immunocompromised individuals from a persistent infectious threat.

## Notes

***Acknowledgments.*** Medical writing and editorial assistance were provided by Ashlea Inan, PhD, and Emily Stackpole, PhD, of MEDiSTRAVA in accordance with Good Publication Practice (GPP 2022) guidelines, funded by Moderna, Inc., and under the direction of the authors.

***Supplement sponsorship.*** This article appears as part of the supplement “COVID-19 Vaccination in the Immunocompromised Population,” sponsored by Moderna, Inc.
